# Intelligent Classification Technique of Hand Motor Imagery Using EEG Beta Rebound Follow-Up Pattern

**DOI:** 10.3390/bios12060384

**Published:** 2022-06-02

**Authors:** Jiachen Wang, Yun-Hsuan Chen, Jie Yang, Mohamad Sawan

**Affiliations:** 1 Center of Excellence in Biomedical Research on Advanced Integrated-on-Chips Neurotechnologies (CenBRAIN Neurotech), School of Engineering, Westlake University, Hangzhou 310024, China; jiachenwang@utexas.edu (J.W.); yangjie@westlake.edu.cn (J.Y.); 2 Institute of Advanced Technology, Westlake Institute for Advanced Study, Hangzhou 310024, China

**Keywords:** wearable electroencephalography, motor imagery, motor execution, beta rebound, brain–machine interface, feature extraction, EEG classification

## Abstract

To apply EEG-based brain-machine interfaces during rehabilitation, separating various tasks during motor imagery (MI) and assimilating MI into motor execution (ME) are needed. Previous studies were focusing on classifying different MI tasks based on complex algorithms. In this paper, we implement intelligent, straightforward, comprehensible, time-efficient, and channel-reduced methods to classify ME versus MI and left- versus right-hand MI. EEG of 30 healthy participants undertaking motional tasks is recorded to investigate two classification tasks. For the first task, we first propose a “follow-up” pattern based on the beta rebound. This method achieves an average classification accuracy of 59.77% ± 11.95% and can be up to 89.47% for finger-crossing. Aside from time-domain information, we map EEG signals to feature space using extraction methods including statistics, wavelet coefficients, average power, sample entropy, and common spatial patterns. To evaluate their practicability, we adopt a support vector machine as an intelligent classifier model and sparse logistic regression as a feature selection technique and achieve 79.51% accuracy. Similar approaches are taken for the second classification reaching 75.22% accuracy. The classifiers we propose show high accuracy and intelligence. The achieved results make our approach highly suitable to be applied to the rehabilitation of paralyzed limbs.

## 1. Introduction

Motor imagery (MI) is a popular method developed to help patients undergoing post-stroke rehabilitation to learn or improve specific motor functions [1]. It is a dynamic state in which patients experience sensations without any actual execution. Studies demonstrate that MI may enhance functional recovery of paralyzed limbs [2], since similar activation sequences occur in the motor cortex during both MI and actual motor execution (ME) [3]. A brain–machine interface (BMI) allows users to interact with the external world through their brain signals instead of their peripheral muscles [4]. Extensive research has been conducted to exploit BMIs for post-stroke rehabilitation, as they assist in the restoration of motional ability [5]. Cincotti et al. demonstrated that, compared with MI alone, rehabilitation training integrated with BMI neurofeedback makes motor areas become more involved, such as by enhancing neuroplasticity in affected regions [6]. Noninvasive electroencephalography (EEG) is a frequently used BMI modality, and one study demonstrated that the majority of stroke patients could use EEG-based MI BMI [7]. One possible application is for evaluating the restoration of physical functions. Until now, the types of assessments commonly used have been time-consuming and can be affected by subjective evaluation.

On the other hand, neurophysiology has revealed that EEG signals experience suppression or enhancement during both MI and ME in the mu and beta frequency bands, which is known as event-related desynchronization (ERD) or event-related synchronization (ERS). Various EEG-based MI BMIs have been developed to detect this phenomenon. The authors of [8] have concluded that beta rebound (beta ERS occurs shortly after movement) is solid and stable without training, promising fast and universal detection. Leeb et al. applied the beta rebound generated by foot MIs as a feature to detect the user’s control intention [9]. Based on beta rebound after foot MIs, Müller-Putz et al. proposed a brain-switch with one-channel EEG [10]. Few studies touch on beta rebound of hand MIs. To the best of our knowledge, no ME versus MI classifications using beta rebound have been reported. Diverse feature extraction methods were proposed to classify left versus right MI [11]. Common spatial pattern (CSP) and its derivatives are proved to result in good accuracy in subsequent classification tasks [12,13,14]. Wang et al. used SampEn to extract features in MI-EEG data and trained classifier, proving its effectiveness [15]. Other techniques, including statistical, wavelet-based, and power-based, were popular in physiological signal processing. Rajdeep et al. extracted 26 features based on these techniques and finished left versus right hand movements classification [16]. These works have already achieved competitive accuracies, but high-dimensional feature vectors can spoil classifier performance, which calls for feature selection to remove redundant features and retain relevant ones [17]. Gu et al. applied sparse logistic regression (SLR) and its derivatives to select features and to estimate their weight parameters for classification, improving the performance of foot MI and acquiring satisfactory results [18]. However, no prior-art publications were found applying SLR to hand MI classification. Foot MI can generate more observable signals and is, therefore, easier to classify [19], but we cannot overlook hand MI as their deftness and indispensable role in daily life.

To assess the restoration objectively, we investigated the difference between ME and MI, intending to assimilate MI into ME in EEG signals with neurofeedback. It is possible to retrain the brain toward becoming more capable of movement, which improves recovery. While the lateral classification (left versus right hand) has achieved high accuracy in upper and lower limbs, few studies have investigated the difference between ME and MI [20,21]. Focusing on power in different frequency bands, Miller et al. confirmed that spatial distribution of neuronal activity during MI mimics that during ME, and its magnitude is ~25% of ME [22]. More detailed distinction should be drawn to ensure a stable detector. Moreover, existing studies of EEG-based MI BMI share the following limitations: (1) few studies have specified the movement or decoded different motors within the same limb [23]; (2) the multichannel EEG signals in these research activities may reduce processing accuracy and speed, while optimal sets of channels are preferable from a practical point of view [24]; (3) little comparative analysis has been conducted to evaluate different feature extraction methods on experimental data set in parallel to determine which ones are preferable [25]; and (4) they feed large quantities of feature vectors directly into classifiers, which will severely limit the accuracy of classifiers [18].

We built a dataset underlining both ME and MI involving delicate motors to address the above-described limitations. This dataset aimed to explore the feasibility of differentiating between ME versus MI and left- versus right-hand MI by optimizing the feature extraction and classification methods. We put forward a stable and straightforward detector of ME and MI based on beta rebound called “follow-up pattern”. We also proposed corresponding methods to address the limitations mentioned before: (1) we reproduced motions that require the engagement of both hands, investigating their application to ME and MI classification; (2) we optimized the number and location of EEG channels to achieve high accuracy with a few channels of EEG-based MI BMI, also proposing a stable and straightforward detector of ME and MI based on beta rebound; (3) we adopted various approaches for extracting features and trained classifiers to validate their utility; and (4) we recognized useful features that improved classification performance with feature-selection techniques. The prepared dataset and analysis methods we proposed can be combined with noninvasive brain stimulation (NIBS) techniques to induce plasticity during post-stroke rehabilitation [26].

The paper is summarized as follows: details of the experiments and the methods of feature selection are described in Section 2; Section 3 illustrates the “follow-up” pattern based on the beta rebound and presents the outcomes of different detection methods; in Section 4 we compare our results with related work; and the conclusions and future work are the subjects of Section 5.

## 2. Materials and Methods

### 2.1. Experiments

**Figure 1:**
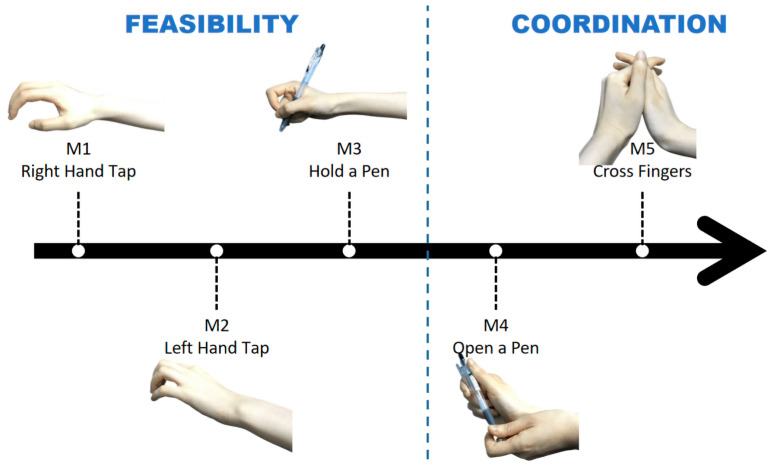
Tasks in the experiment. M1–M3 were to examine the feasibility, and M4–M5 were set for coordination. M1: move specific right fingers according to the auditory code; M2: move specific left fingers according to the auditory code; M3: make the gesture of holding a pen and ready to write; M4: unscrew the pen; M5: fingers of both hands cross over each other.

**Figure 2:**
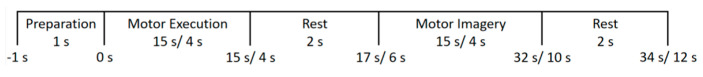
Timing paradigm of one trial: the duration of motor execution can be 15 s (tapping each finger for 3 s) or 4 s (other tasks); the endpoint of motor imagery depends on the participant’s self-regarded “completion”. The overall time course is estimated and denoted at the bottom. It can vary between subjects and tasks.

In our research, 30 healthy individuals (15 males, 15 females; aged 20–35 years, mean ± SD: 24.26 ± 3.46; 29 are right-handed) volunteered. All participants provided written informed consent in accordance with the Declaration of Helsinki before the experiment, which was approved by the ethical committee of Westlake University, Hangzhou, China (approval ID: 20191023swan001). All participants received CNY 100 as an inconvenience allowance. Participants were required to make movements based on auditory stimuli, undertaking the following actions: finger tapping, holding a pen, opening a pen, crossing fingers, and moving the arm, as shown in Figure 1. The tasks were set to examine the feasibility (whether joints and hard tissues constrain the freedom of movement) and coordination (all fingers should work in coordination to serve a common purpose, i.e., participants place their hands flat on the table in a comfortable way, while each finger start to orchestrate the required movement after coordination stimuli) of both-hand motion. Each task included five trials for ME and five trials for MI. Each trial was followed by a 2 s rest time. The timing paradigm of a single trial is shown in Figure 2.

### 2.2. EEG System

**Figure 3:**
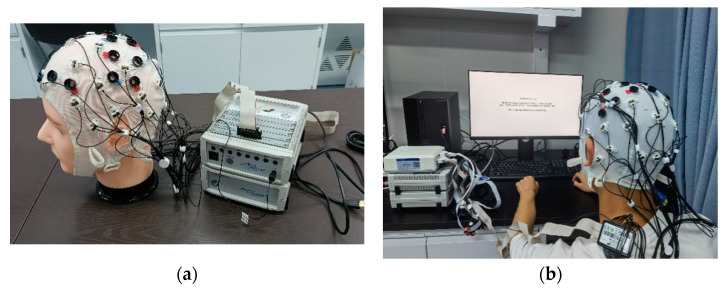
(**a**) The EEG system used in our study; (**b**) the recording scene: a participant is following the instructions showing on the screen when the EEG signals are recorded.

The EEG system examined in this study was the *Brain Products actiCHamp Plus* (EEG signal amplifier) and actiCAP slim (active EEG electrodes) provided by Brain Products GmbH, Munich, Germany, as shown in Figure 3. Thirty-two active electrodes including a reference electrode and a ground electrode were introduced to the system. These electrodes can be placed onto three fabric caps (54–56 cm, 56–58 cm, or 58–60 cm), catering for participants’ head circumstances. A chin belt was attached to each cap to achieve better fixation and maintain electrodes’ position on the scalp. In total, 32 possible electrode positions arranged under a 10–20 international standard system were marked on each cap.

Before each experiment, a disinfectant wipe was applied to the electrodes. When finished, electrodes and caps were carefully cleaned from gels. These practices can effectively prevent crosstalk between channels induced by resting gels and enhance connectivity by removing dust and particles within the system.

### 2.3. Data Recording and Preprocessing

**Figure 4:**
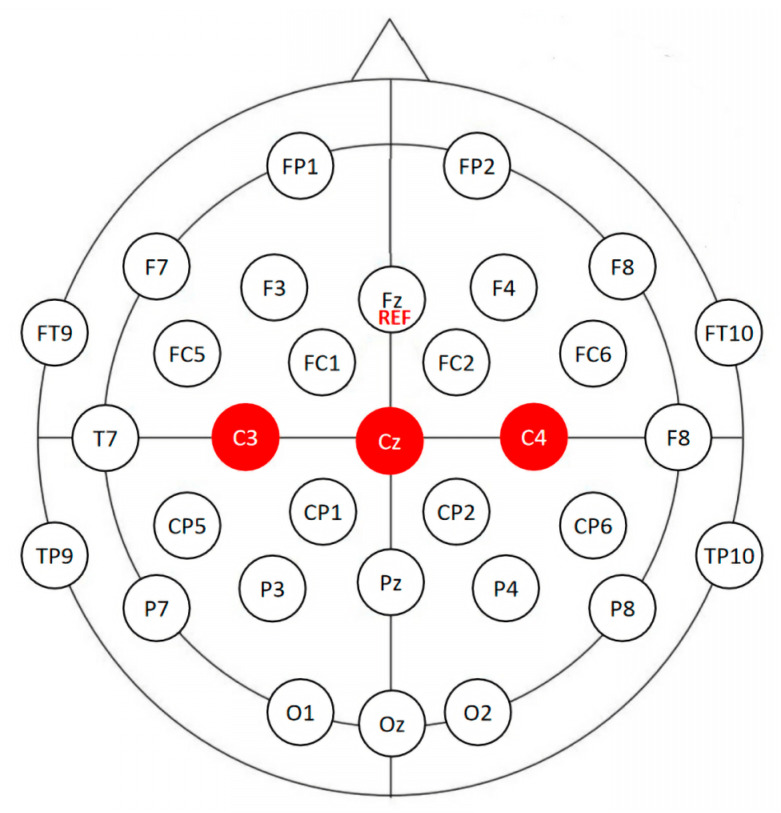
The 32-channel EEG recording montage used in our experiments. Channels C3, C4, and Cz are in the mid-central area, marked as red circles. REF denotes that Fz is the reference electrode.

EEG signals were recorded with Ag/AgCl electrodes in a 32-channel cap arranged under a 10–20 international standard system (Brain Products, Inc, Gilching, Germany). The central frontal electrode (Fz) served as a reference to a common ground, and the impedance was controlled to be lower than 10 kΩ. The EEG data were recorded with a sampling rate of 1000 Hz. The montage used in our experiment is shown in Figure 4.

Preprocessing included the following procedures: removal of bad channels (channels that coupled noise or had irregular power spectra) or segments, re-referencing to a common average (common average reference is the average electrical activity measured across all scalp channels, re-referencing is conducted by subtracting it from each channel.), filter from 1 to 60 Hz and a 50 Hz notch filter (the interferences from mainline power are removed by the 50 Hz notch filter and EEG signals at 1 to 60 Hz contain most useful information for our applications.), independent component analysis (ICA), epoch extraction, and baseline correction. In the two sets (ME and MI) of preprocessed EEG data, a total of 812 epochs were generated. According to [27], the primary motor cortex (PMC) region, where channels C3, C4, and Cz are located, includes more signals for higher classification performance than other brain areas. We adopted these channels in subsequent analysis to shorten the experiment’s preparation time and to reduce the computation load to realize a BMI that requires less input information. We attempted to classify ME and MI with a single channel, Cz, and EEG signals from 19 subjects (with good-quality Cz) were applied. While the classification of left- versus right-hand MI requires more lateral information, 10 participants (with good-quality C3, C4, and Cz) were selected for this task.

### 2.4. Event-Related Desynchronization/Synchronization Analysis

The definition of the ratio ERD/ERS can be formulated as:

(1) $$ERD/ERS_{i}=\frac{A_{i}-R}{R}\times 100\%$$

where $A_{i}$ is the average power of ith sample over all the trials and *R* is the average power in the reference interval [28]. The value is defined as ERS when $A_{i}$ is greater than R.

*ERD/ERS* values ranging from 13 to 40 Hz were computed to observe beta rebound. *ERD/ERS* values were considered significant with 95% confidence by adopting a bootstrap *t*-test.

### 2.5. Feature Extraction

Sample entropy (SampEn) evaluates the complexity and regularity of time-series data, measuring the unpredictability of fluctuations in physiological signals [29]. Let $x\left(T\right)$ denote the EEG time series, where T represents the length. To calculate SampEn, we should determine the series of vectors length, m, beforehand:

(2) $$X\left(i\right)=\left[x\left(i\right),x\left(i+m-1\right)\right],\;i=1,2,\ldots ,\left(T-m+1\right)$$

Similar tolerance r controls the number of vector $X\left(j\right)$ such that:

(3) $$N^{m}\left(i\right)=card\left\{X\left(j\right)|dist^{m}\left\{X\left(i\right),X\left(j\right)\right\}<r\right\}$$

where $dist^{m}\left\{X\left(i\right),X\left(j\right)\right\}$ is defined as the most considerable absolute difference between each scalar component.

(4) $$B^{m}\left(r\right)=\frac{1}{{\left(T-m+1\right)}^{2}}\overset{T-m+1}{\underset{i=1}{\sum }}N^{m}\left(i\right)$$

SampEn is then defined as the negative logarithm of $\frac{B^{m+1}\left(r\right)}{B^{m}\left(r\right)}$.

Here, we computed the SampEn of the Cz, C3, and C4 channels from 10 participants, with a series of vector lengths m=2 based on both raw EEG data (r=1.0∗SD, where SD denotes the standard deviation) and ERD/ERS data (r=0.1∗SD). These values were chosen by enumeration and while examining their performance when training classifiers.

The common spatial pattern (CSP) is an advanced algorithm based on principal component analysis (PCA), and it has been successfully applied to brain–computer interfaces [30]. CSP filters EEG signals of two classes to make a clear distinction between them. The feature vectors $f_{i}\;$ are defined by Equation (5):

(5) $$f_{i}=\log\left(\frac{\mathrm{var}\left(Y_{i}\right)}{\sum_{k=1}^{k=2}\log\left(\mathrm{var}\left(Y_{k}\right)\right)}\right),\;i=1,2$$

where var represents the variance of a specific sequence and $Y_{i}$ denotes the corresponding column of CSP-filtered data.

Statistical feature vectors include standard deviation of raw signals and the mean of the absolute values of both the first and second differences of the raw and standardized signal.

We applied Daubechies mother wavelets of order 4 (db4) to analyze the raw EEG data, and the detailed coefficients at level 3 were used to extract features. The related feature vectors were wavelet root mean square (RMS), energy (ENG), and entropy (ENT) [31].

Average power within a specific frequency band was estimated by the average power spectrum density (PSD). The average band power is defined as the power ratio in a specific frequency band to total power. We applied the Welch approach to estimate the PSD with a Hamming window. We performed a PSD estimation on two rhythms, alpha (8–12 Hz) and beta (13–40 Hz).

Details of the feature vectors applied to the classification of ME versus MI, and the classification of laterality in MI, are listed in Table 1 and Table 2, respectively.

### 2.6. Support Vector Machine Classifier

With statistical learning, a support vector machine (SVM) can tackle problems involving small training sets and nonlinear relationships in classification tasks [32]. SVM is used to optimize a hypersurface to separate different classes and to enlarge the distances between them. The MATLAB function *fitcsvm* was applied to train and cross-validates SVM models for our classification tasks.

### 2.7. Feature Selection

For neuroimaging data, where the training set is small while the feature dimensionality is large, logistic regression is not applicable. In sparse logistic regression (SLR), every weight parameter has its own adjustable variance referred to as relevance parameters, controlling the possible range of the corresponding weight parameters. The weight parameters are estimated as the marginal posterior mean, which can be estimated by variational Bayesian approximation (SLR-VAR) or Laplace approximation (SLR-LAP). The L1-norm-SLR with a Laplace approximation (L1-SLR-LAP) and the component-wise implementation (L1-SLR-COMP) were also investigated in this study [18].

## 3. Results

### 3.1. “Follow-Up” Pattern

**Figure 5:**
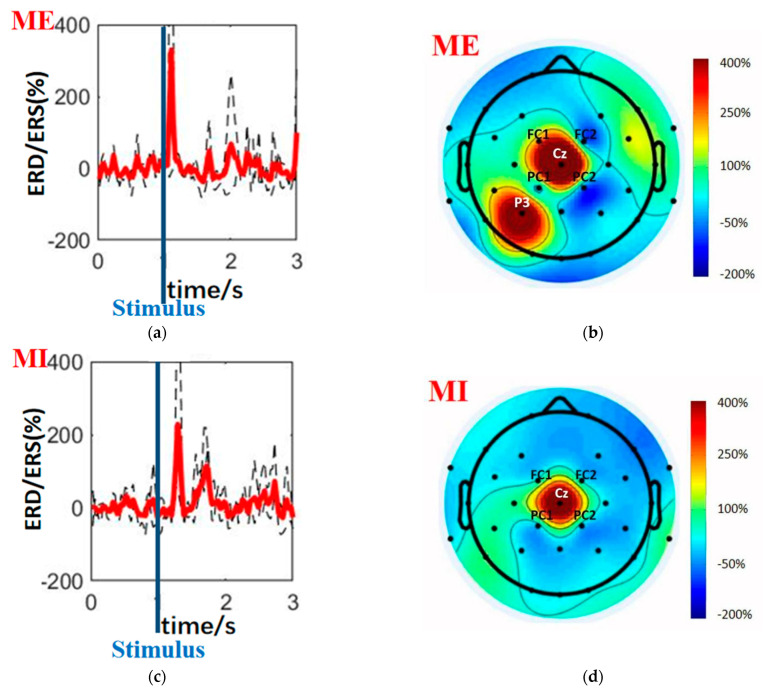
“Follow-up” pattern based on the beta rebound works as an indicator of ME and MI in amplitude, latency, duration, and distribution. An example of the time courses and topo-plots of ME (parts (**a**,**b**)) and MI (parts (**c**,**d**)) event-related desynchronization/synchronization (ERD/ERS) during the motion Tap: Right Finger 1. The bold red lines are the average across five subjects, while the dashed lines are the individuals’ ERD/ERS time courses at channel Cz. “Stimulus” marks the time when subjects hear the auditory instructions. Topo-plots are from subject S01 for motion Tap: Right Finger 1 at the time when the beta rebound is the most remarkable (ME: 1.624 s; MI: 1.818 s). Black dots mark the locations of electrodes. ERS is in red while ERD is in blue. Note that parts (**a**,**c**) are based on a part of our whole dataset (26.32%) to make the time courses more explicit for demonstration.

Beta rebound is a stable phenomenon that occurs several seconds after ME or MI. As shown in Figure 5, the beta rebound is the beta ERS (refer to Formula (1)) that occurs within 1 s after a stimulus (represented as blue lines). It can be observed in participants with little or no training. Taking advantage of this primitive and perceptible reaction, we proposed a method based on the beta rebound in the time-domain signals to discriminate between ME and MI that requires a light computational load and little pre-training. This time-domain “follow-up” pattern helps therapists gain information from the beta rebound in real time, evaluate the performance of paralyzed patients, and then guide and rectify their MI tasks. With proper training, the beta rebound can offer novel targets for therapeutic interventions [33].

Figure 5 demonstrates the difference between ME and MI in both the time and spatial domains. In the time domain, ME and MI have a distinction in amplitude, time delay, and latency. Figure 5a (ME) and Figure 5c (MI) illustrate it as ERD/ERS time courses during the same finger-tapping movement (motion Tap: Right Finger 1), with dashed lines from five different individuals while bold red lines delineating the average time course across these subjects. Beta rebounds are represented as peaks in these lines. “Stimulus” marks the time when subjects hear the auditory instructions. Compared with ME, the beta rebounds of MI have smaller amplitude, appear later after stimulus, and last longer. In the spatial domain, ME and MI have different topographic distributions. Figure 5b (ME) and Figure 5d (MI) demonstrate it with topo-plots (topographic maps of EEG fields in a circular 2D view looking down at the top of the head) depicting ERD/ERS distribution. These topo-plots are from subject S01 for motion Tap: Right Finger 1 at the time when the beta rebound is the most remarkable (ME: 1.624 s; MI: 1.818 s). Black dots mark the locations of electrodes. ERS is in red while ERD is in blue. During the MI task (Figure 5d), the beta rebound was constrained within the mid-central areas (channel Cz). In contrast, the rebound of ME (Figure 5b) had a more enormous scope of influence, affecting adjacent electrodes (channels Cz, FC1, FC2, PC1, PC2, and P3). Cz and the surrounded channels are related to sensorimotor cortex, which accounts for the peak at the mid-central areas. The other peak at channel P3 may attribute to the touch sensation function of parietal lobe, which only occur during ME. To conclude, in the time domain, there is a high probability that beta rebounds of lower intensity, higher latency, and longer duration indicate MIs instead of MEs; in the spatial domain, if beta rebounds mainly affect channel Cz, it will most likely represent MIs.

**Figure 6:**
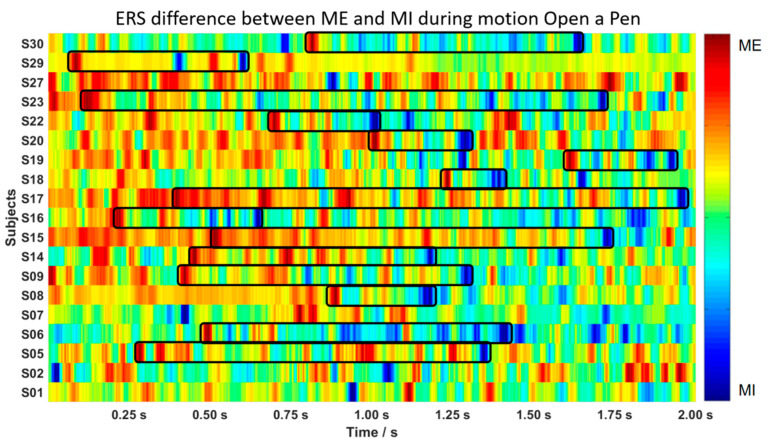
Color map of the differences between ME and MI tasks during the motion, open a pen. Red blocks show ERS during ME, while blue blocks represent ERS during MI. In most cases, a “follow-up” pattern—a red block followed by a blue block—can be observed, marked by the black frames.

We computed the difference in the ERD/ERS values between ME and MI by subtracting the signals of each motion recorded from each subject. The results of the subtracted signals during M4 (Figure 1), open a pen, are illustrated by a pseudo-color map in Figure 6, with the x-axis representing post-stimuli time and the y-axis representing subjects. Each pixel indicates the intensity by color, where red denotes the beta rebound of ME, and blue denotes the beta rebound of MI. As marked by black frames (as an example) in Figure 6, most participants’ data observes the “follow-up” patterns. The “follow-up” pattern implies that the beta rebound of ME can occur faster than that of MI, following the difference described above in Figure 5. We marked all the peaks in the ERS series and counted all the “follow-up” phenomena across subjects and across motional tasks. The results are shown in Table 3 and Table 4. Table 3 defines the percentage as the ratio of motions that displayed “follow-up” patterns. Some subjects, e.g., S06 and S18, achieved high accuracy under this criterion, which reflected the variation across subjects: some subjects are more adapted to imaginary tasks than others. Throughout all the motions listed in Table 4, opening pens and finger-crossing were distinctive compared to the others, and they are both motions designated to examine coordination in movement. The motions that require both hands’ involvement and synchronization have more significant potential to be applied in the evaluation system of a rehabilitation process. The parameters of beta rebound (amplitude and time) of ME and MI tasks can be distinguished more obviously.

Based on the findings mentioned above, we can conclude how to identify ME and MI with beta rebound at channel Cz in the time-domain: compared with ME, beta rebounds of MI have smaller amplitude, appear later after stimulus, and last longer.

### 3.2. ME versus MI Classification

We used feature vectors in Table 1 to train SVM and adopted hyperparameter optimization during training to search for kernel functions and related parameters to induce the best performance. Such procedures achieved a classification accuracy of 78.57%. We drew the scatter plots and found that power-related features may perform better in EEG classification tasks. We selected those four power-based feature vectors to describe the data set and trained the SVM again. The overall accuracy improved slightly to 79.51%, but the dimension of features was reduced, which will mitigate the computational load. Additionally, this indicates that excessive large feature vectors may not necessarily lead to higher accuracy in SVM classification tasks. Feature selection methods can be applied in training classification models, which enlightened us about resorting to SLR in left- versus right-hand MI classification tasks, as described in the following paragraphs.

### 3.3. Left—Versus Right-Hand Motor Imagery Classification

We adopted features in Table 2 to train a classifier that may facilitate SVM task in a higher dimensionality. The accuracy was only 62%, which was even lower than when sample entropy feature vectors were applied alone. This phenomenon warned us there were some redundant feature vectors in the SVM training data that spoiled the overall result.

**Figure 7:**
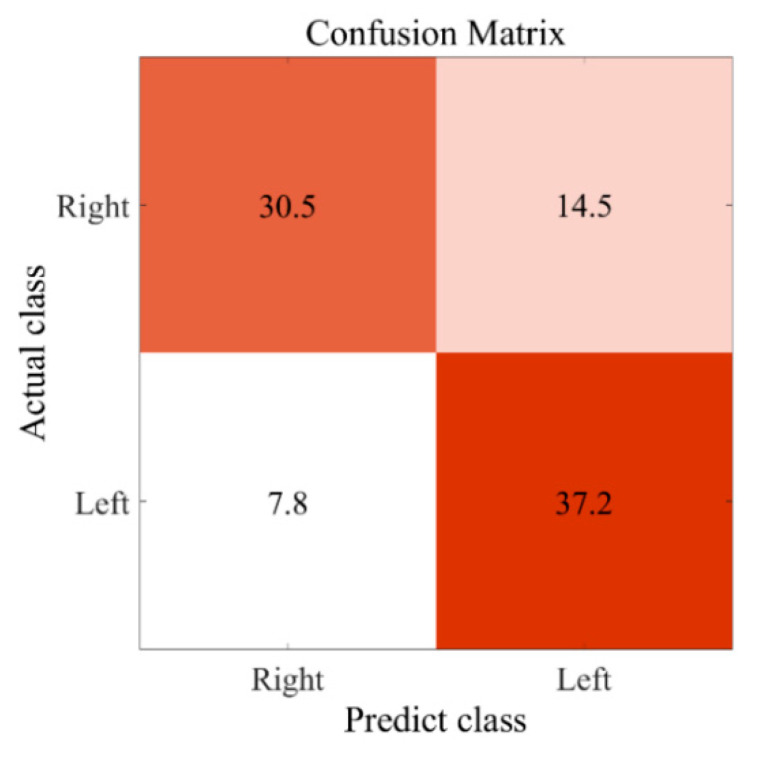
Confusion matrix of the “L1-SLR-LAP” classifier to distinguish the MI of left- and right-hands.

We adopted different derivatives of SLR to select features and to calculate weights. The number of features left and the corresponding accuracies are shown in Table 5. Among all the models adopted, L1-SLR-LAP, which applied Laplacian approximation and L1-norm in SLR learning, attained the best performance. The accuracy of L1-SLR-LAP is 75.22%, and the corresponding confusion matrix is displayed in Figure 7. Note that the values here are the average number of 10-fold cross-validation. Higher accuracy was achieved in left-hand MI. Forty-two feature vectors were left after the selection procedure in L1-SLR-LAP. By checking their weights, we found that power features and SampEn displayed distinctive weights in the remaining vectors, which indicated that they were primary factors in the classification task.

### 3.4. Comparison and Analyses of Classification Accuracies

Previous studies of MI classification tasks generate interesting classification accuracies, based on different datasets, models and techniques. Table 6 compares the classification results of left- and right-hand MI among the proposed dataset and other datasets. It is important to note that the accuracy of our proposed method is obtained through group-level classification, while in other works, classifiers are trained in a subject-specific manner. Group-level classifications will reduce training sessions and be more applicable to patients, as elucidated in Section 4. Using the same EEG channels and classifier models as the ones we proposed, Malan et al. [34] suggested a novel feature selection algorithm, regularized neighborhood component analysis (RNCA), which outperformed other conventional feature selection techniques. The diverse parameters of RNCA increase its computational burden, while SLR is lighter. The dimension of features in [35] was relatively low, so the accuracy is comparable without feature selection. We achieved a similar accuracy with fewer EEG channels, which can lighten the workload of experiment and computation. Accuracies in [36] seem lower than other studies, which may verify that SVM is more preferable in such contexts.

## 4. Discussion

We applied a single neuroimaging modality, EEG, in the present study. EEG has a high temporal resolution and can produce good performance in BMI [18]. Other modalities have been explored, e.g., functional magnetic resonance imaging (fMRI) [37], functional near-infrared spectroscopy (fNIRS) [38], magnetoencephalography (MEG) [39], and electrical impedance tomography (EIT) [40]. Due to portability, non-invasiveness, and cost-effectiveness, EEG and fNIRS have an advantage in natural environment applications [41]. In terms of classification accuracy, EEG-based BMI outperforms fNIRS-based BMI [24]. Recent progress of hybrid EEG-fNIRS in BMI demonstrates great potential because data with complementary spatiotemporal resolution can exhibit synergistic effects, bringing about insights into crucial brain processes and structures.

Most reported EEG-based BMI systems can be categorized into one of three paradigms: motor imagery (MI), event-related potential (ERP), and steady-state visually evoked potential (SSVEP). We adopted MI, although successful cases of other paradigms have been proposed, such as P300 ERP [42], SSVEP [43], spatial attention [44], selective attention [45], mental arithmetic [46], action observation [47], late positive potential (LPP) [48], etc. With no need for external stimuli, motor imagery tasks are self-paced, simple, and stable. Our results validate its utility in EEG-based BMI.

The “follow-up” pattern we proposed is based on beta rebound. The mid-centrally located beta rebounds reveal electrophysiological correlates of synchronized “resetting” from overlapping brain networks. The occurrence of beta rebound depends heavily on the types of MI. Our study found that motors with more fingers involved can lead to better results. It can probably be explained by the superposition effect of MI, i.e., the neural activities triggered by hand MI can be interpreted as the summation of the activities invoked by simple finger MIs, which is validated in [49]. The variation is not limited to upper limbs. According to [19], most subjects displayed beta ERS during foot MI, while tongue MI induced no beta rebound in any subject. Luckily, even if there is only a slight laterality difference in the subject, improved BMI control accuracy can be achieved through visual feedback [50].

It is common practice to extract features based on statistical properties, wavelet coefficients, and average power [16]. In this work, we compared the features generated by these above-mentioned principles and SampEn and CSP. Our results show that power features and SampEn play a dominant role in classification tasks. Other innovative methods were proposed for extraction to solve MI classification tasks. Functional brain networks are being widely applied to extract extra features, delineating the interactions between each pair of electrodes [51].

Despite its popularity, SVM is not the only classifier model that can succeed in MI-EEG classification tasks. To evaluate their performance in EEG-based MI BMI, a comparative analysis of five classifiers, SVM, k-NN, naïve Bayes, decision tree, and logistic regression, was conducted in [52] and it concluded that SVM, logistic regression, and naïve Bayes outperformed the others in accuracy. Recently, with automatic end-to-end learning, deep learning (DL) is competent in this context, simplifying processing pipelines; hence, improved performance can be achieved [53].

Instead of feeding large quantities of feature vectors directly into classifiers, a three-feature selection method SLR was applied to lower the dimensionality of features in this work, with the intention of improving classification accuracy. Gu et al. applied a similar method in foot MI classification tasks, with the most remarkable accuracy of 75.33% achieved by SLR-variational approximation (SLR-VAR) [18]. Rejer et al. compared different methods of feature selection on the left- and right-hand MI [54]. Feature selection may also help discover new patterns of brain behavior and invent new explanations for neural pathways. μ-rhythm was suggested to reflect the translation of hearing an instruction into performing the required action, which is well in line with the feature selection results [55].

It is important to note that only a small portion of channels was used in subsequent analysis—to be specific, a single channel (Cz) in ME versus MI classification and three channels (Cz, C3, and C4) in left- versus right-hand MI classification. These channels have been proven to induce better classification results [24,27]. In previous EEG-based MI BMI, a large portion used many EEG channels. Thirty-two EEG electrodes are used in [56] and they achieve a classification accuracy of 59.65%. We successfully classified ME versus MI by 79.51% with one channel (Cz) and left- versus right-hand MI by 75.22% with three channels (Cz, C3, and C4). In general, we achieved better performance with less data, which can alleviate the computation load and reduce experiment preparation time.

Most classifiers in EEG-based BMI studies are trained in a subject-specific manner, which can decode intention from a specific patient based on his own signal features [18,57]. This manner demands laborious training for subjects and repetitive signal processing to ensure solid results. Moreover, it is also infeasible for physically disabled patients to provide these training data. Here, we trained classifiers with population-level features obtained from different subjects and gained competitive performance. It demonstrates excellent potential for simplified application, since real-time EEG signals can be acquired from patients without training and compared with the existing training sets.

Despite the group-level classification, our accuracy is still comparable. A possible merit lies in our carefully selected features for training SVM. In classification between ME and MI, we selected power-based features manually, which improved the accuracy minutely but reduced the feature dimension greatly. In classification between left- and right-hand MI, we adopted SLR to abrogate redundant feature vectors, so the corresponding accuracy increased by 13.22%. Admittedly, we did not reach perfect accuracy, but this appears reasonable given that untrained subjects can be unaffected by BMI protocols. The term “BMI illiteracy” was coined for this non-negligible portion of users, which is estimated at 15% to 30% [58]. The BMI illiteracy rate matches our classification results.

Our research explored the feasibility of EEG for evaluating post-stroke recovery. Previous work cross-validates the efficacy of EEG signals with other assessments, such as motor functions and activities of daily living (ADL), Fugl–Meyer assessment (FMA) scores, and the modified Ashworth scale (MAS). Based upon this fact and our results, we further propose a prospective for EEG assessment, wherein therapists record EEG signals from patients during rehabilitative MI tasks, then label them with classifiers trained by group level training sets and provide real-time feedback to make patients aware of the similarity between their neural activities and the correct ones.

Admittedly, the current study embodies some limitations. The experimental procedure can be modified, allowing subjects to repeat MI within specific time slots. Such modification will not only facilitate analysis but also induce more detectable signals. Moreover, our analysis was based on sensor-level techniques, while the volume conduction effect calls for source-level analysis, which would map EEG signals to cortical areas. The “follow-up” pattern we generalized still improves classification accuracy, so more detailed characteristics can be drawn from it.

## 5. Conclusions

EEG-based MI BMI has great potential in evaluating post-stroke rehabilitation. However, present assessments suffer from low efficiency and lack of objectiveness, and few related studies underline the difference between ME and MI. In this work, we proposed a dataset and corresponding analysis methods to classify both ME versus MI and left- versus right-hand MI tasks, which can induce plasticity during restoration. This study put forward a stable and straightforward detector of ME and MI based on beta rebound, investigated extracted feature vectors, and applied SVM with SLR to classification. The conclusions are summarized as follows:

“Follow-up” pattern based on the beta rebound is a stable indicator of ME and MI. Compared with ME, the beta rebounds of MI have smaller amplitude, appear later after stimulus, and last longer. The phenomenon is most significant at channel Cz. Such characteristics defined the “follow-up” pattern. Its occurrence is 59.77% ± 11.95% among all subjects, and motors with more fingers involved can generate better results (finger-crossing: 89.47%).

The ME versus MI classification accuracy is 79.51% with power-based features and SVM. We extracted 13 features with statistic, wavelet-based, and power-based methods. SVM generated a classification accuracy of 78.57% with these feature vectors. After examining the support vectors, features fed into SVM were pruned back to four power-based ones, while the accuracy increased.

The left- versus right-hand MI classification accuracy is 75.22% with SVM and L1-SLR-LAP. We extracted 59 features with statistic, wavelet-based, power-based, SampEn, and CSP methods. We compared the performance of different derivatives of SLR and found out that L1-SLR-LAP win over others with 42 feature vectors left. We concluded that power-based features and SampEn displayed distinctive weights in the remaining vectors.

Therefore, this work demonstrates an innovative approach that can be used for evaluating the rehabilitation results of MI BMI with neurofeedback. In future work, we will focus on the back-end design of the system and explore the addition of NIBS as an adjunct therapy. BMI+NIBS interventions could inform patients and therapists about real-time MI performance and enhance rehabilitation with additional clinical gains.

## Figures and Tables

**Table 1:** Feature vectors classifying motor execution (ME) versus motor imagery (MI).

Feature Vectors	Size (No. of Trials × No. of Features)
Statistical Features	532 × 6
Wavelet-based Features	532 × 3
Power Features	532 × 4
Total	532 × 13

**Table 2:** Feature vectors classifying left versus right hand.

Feature Vectors	Size (No. of Trials × No. of Features)
Statistical Features	100 × 18
Wavelet-based Features	100 × 9
Power Features	100 × 24
SampEn	100 × 6
CSP	100 × 2
Total	100 × 59

**Table 3:** Percentage of “follow-up” pattern in subjects at Cz among all motions.

Subjects	Percentage (%)	Subjects	Percentage (%)
S01	50.00	S17	57.14
S02	50.00	S18	85.71
S05	57.14	S19	42.86
S06	78.57	S20	57.14
S07	42.86	S22	57.14
S08	64.29	S23	64.29
S09	50.00	S27	64.29
S14	71.43	S29	71.43
S15	71.43	S30	50.00
S16	50.00	Mean ± SD	59.77 ± 11.95

**Table 4:** Percentage of “follow-up” pattern in motions at Cz among all subjects.

Motions	Percentage (%)	Motions	Percentage (%)
Tap: Right Finger 1	57.89	Tap: Left Finger 4	57.89
Tap: Right Finger 2	36.84	Tap: Left Finger 5	42.10
Tap: Right Finger 3	57.89	Hold a Pen	63.16
Tap: Right Finger 4	63.16	Open a Pen	84.21
Tap: Right Finger 5	52.63	Finger-crossing	89.47
Tap: Left Finger 1	68.42	Arm Movement	52.63
Tap: Left Finger 2	52.63	Mean ± SD	59.77 ± 13.58
Tap: Left Finger 3	57.89		

**Table 5:** Accuracy of different classifiers used on our EEG data.

Models	Features Left	Accuracy
SVM	59	62.00%
SLR-LAP	2	57.78%
SLR-VAR	9	50.22%
L1-SLR-LAP	42	75.22%
L1-SLR-COMP	35	58.67%

**Table 6:** Comparison of classification accuracies among different datasets and methods.

Authors	EEG Channels	Participants	Feature Extraction	Classifiers	Feature Selection	Average Accuracy
This work	3	10	Statistics, Wavelet Coefficients, Average Power, SampEn, CSP	SVM	L1-SLR-LAP	75.2%
Malan et al., 2019 [34]	3	10	DTCWT	SVM	GA	78.9%
					PCA	64.3%
					ReliefF	75.7%
					RNCA	80.7%
Tang et al., 2017 [35]	28	2	Power spectrum	SVM	-	77.2%
Voinas et al., 2022 [36]	16	6	WPD+HOS	RF	-	71.0%
			CSP			66.0%
			Filter Bank CSP			69.0%

CSP: common spatial pattern; DTCWT: dual-tree complex wavelet transform; GA: genetic algorithm; L1-SLR-LAP: L1-norm-SLR with a Laplace approximation; PCA: principal component analysis; RF: Random Forest; RNCA: regularized neighborhood component analysis; SampEn: sample entropy; SVM: support vector machine; WPD+HOS: wavelet packet decomposition combined with higher order statistics.

## Data Availability

The data presented in this study are available from the corresponding authors, Y.-H.C. and M.S., upon request. The data are not publicly available because they contain information that could compromise the privacy of research participants.
